# *Jasada bhasma*, a Zinc-Based Ayurvedic Preparation: Contemporary Evidence of Antidiabetic Activity Inspires Development of a Nanomedicine

**DOI:** 10.1155/2015/193156

**Published:** 2015-03-18

**Authors:** Rinku D. Umrani, Kishore M. Paknikar

**Affiliations:** Centre for Nanobioscience, Agharkar Research Institute, G. G. Agarkar Road, Pune Maharashtra 411004, India

## Abstract

The roles of metals in human physiology are well established. It is also known that many metals are required in trace amounts for normal metabolism and their deficiency leads to diseases. In Ayurveda, metal-based preparations, that is, *bhasmas*, are indicated for the treatment of several diseases. Standard textbooks of Ayurveda recommend *Jasada bhasma* (zinc based *bhasma*) as the treatment of choice for diabetes. Modern medicine also recognizes the important role of zinc in glucose homeostasis. Yet, studies that validate the use of *Jasada bhasma* are few and uncomprehensive. There is an imminent need for a systematic study on physicochemical characterization, pharmacological efficacy, and toxicity assessment of several *bhasma* preparations to generate scientific evidence of their utility and safety. Interestingly, recent studies suggest that *bhasmas* comprise submicronic particles or nanoparticles. Thus a *bhasma*-inspired new drug discovery approach could emerge in which several metal based nanomedicines could be developed. This would help in utilizing the age old, time-tested wisdom of Ayurveda in modern medicine. One such study on antidiabetic activity of *Jasada bhasma* and the corresponding new drug, namely, zinc oxide nanoparticles, is briefly discussed, as an example.

## 1. Diabetes

Diabetes mellitus is a metabolic disorder manifested by the presence of hyperglycemia, that is, fasting glucose levels >140 mg/dL and postprandial glucose levels >200 mg/dL. It is a heterogeneous group of disorders influenced by age, genetic composition, and environmental factors. The pancreatic *β*-cells and their secretory product, namely, insulin, are central in the pathophysiology of diabetes [1]. Based on the insulin levels and pancreatic function, two main types of diabetes are recognized. Insulin-dependent diabetes mellitus (IDDM) or type 1 diabetes is due to autoimmune destruction of the insulin-producing pancreatic *β*-cells resulting in an absolute deficiency of insulin. Therefore, type 1 diabetic patients need exogenous insulin for survival [2]. In noninsulin-dependent diabetes mellitus (NIDDM) or type 2 diabetes, muscle, liver, and fat cells become “resistant” to the actions of insulin. Also, the compensatory mechanisms that are activated in the *β*-cells to secrete more insulin are not sufficient to maintain blood glucose levels within a normal physiological range [1]. Besides type 1 and type 2 diabetes, several other forms of diabetes such as “maturity onset diabetes of the young” (MODY), “gestational diabetes,” and “maternally inherited mitochondrial diabetes” (MIMD) are reported. However, the incidence of type 2 diabetes is the highest (~90%) followed by type 1 diabetes (~9%) among the diabetic population [2].

Currently, the only therapy for type 1 diabetes is administration of insulin and/or analogues (which differ from human insulin by one or two amino acids). Patient discomfort due to multiple injections a day and weight gain are major demerits. Considerable efforts have been made for the development of oral insulin for better patient compliance. However, such options are not yet available in the market and insulin remains the mainstay of treatment of type 1 diabetes.

Several oral antidiabetic agents are clinically used for the treatment of type 2 diabetes (Table 1). Life style and dietary changes are also recommended in early stage of the disease. Other than the listed agents, insulin and analogues are used in the late stage of type 2 diabetes, especially in patients with poor glycemic control. However, these therapies are associated with several side effects based on their mechanism of action [3–5], summarized in Table 1.

## 2. Need for Newer Antidiabetic Drugs

Most of the antidiabetic agents cannot be used as a single therapy and are used in combination with each other or with insulin, increasing the treatment cost. Despite available polytherapy, current unmet needs are

- enhanced insulin secretion without the risk of hypoglycemia,
- increased insulin sensitivity without body weight gain,
- improvement in dyslipidemia that coexists with diabetes,
- preservation of pancreatic beta cell action and delayed beta cell failure,
- delayed development of diabetes related complications, namely, retinopathy, nephropathy, neuropathy, and cardiomyopathy.

A single, cost-effective, oral, antidiabetic treatment with minimal side effects is the need of the day. As an important part of the continued research on developing newer antidiabetic agents, several metals are being investigated for beneficial effects in both type 1 and type 2 diabetes and associated complications. These include vanadium, chromium, magnesium, selenium, cobalt, zinc, tungsten, and molybdenum. Of these metals, zinc is of particular interest due to its pleiotropic role as discussed below.

## 3. Role of Zinc in Glucose Metabolism

Zinc is an essential micronutrient, found in all tissues of the body, 95% of it being intracellular [6]. Being a cofactor of more than 300 enzymes, zinc is involved in all cellular functions including signal transduction, transcription, and replication [7]. Zinc is also a cofactor in DNA, RNA, and protein synthesis and influences gene expression through transcription factors [8]. Zinc also plays a role in growth, development, apoptosis, immune function, reproduction, maintenance of vision, protein digestion, blood clotting, bone metabolism, and carbohydrate metabolism.

**Figure 1:**
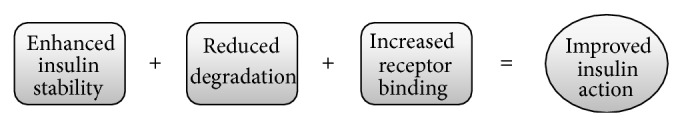
A schematic of the reported mechanisms by which zinc improves insulin action [9–13].

The pancreas is the site of insulin synthesis, storage, and secretion, and zinc is involved in each of these processes [9]. Even before any evidence of a relationship between zinc and insulin existed, it was known that addition of zinc extended insulin's duration of action [10]. As early as 1930's, zinc was known to be important for the integrity of the crystalline structure of insulin [11]. In the presence of zinc within the beta cell, insulin monomers assemble to a dimeric form for storage and secretion as the zinc crystal. Dimeric insulin assembles further into a hexamer that is relatively more stable form of insulin [12]. Zinc not only prevents the degradation of insulin hexamers but also improves the binding of insulin to its receptors and inhibits degradation by liver plasma membranes [13]. These reported mechanisms might be working together to improve insulin action (Figure 1).

Insulin mimetic actions of zinc are also known, reported as early as 1980, where zinc chloride stimulated lipogenesis in rat adipocytes [14]. It is now known that these insulin mimetic effects in adipocytes are through a complex interplay of improved insulin signaling, increased glucose transport, phosphodiesterase activation, and inhibition of free fatty acids release [15]. Haase and Maret [16] reported PTP1B inhibition by zinc, thus enhancing insulin signaling. Further, zinc enhances insulin signaling by increased insulin receptor tyrosine phosphorylation, enhanced PI3K activity, and inhibition of GSK3 [17].

Zinc also has beneficial effects on glucose metabolism. Inhibition of intestinal glucose absorption by zinc has been reported [18]. By inhibiting fructose 1,6-bisphosphatase [13], zinc favors glycolysis as opposed to gluconeogenesis in the cell. Zinc induces the translocation of glucose transporters (GLUT4) to plasma membrane in adipocytes [19, 20], thus increasing glucose uptake and reducing blood glucose levels.

In the adipocytes, insulin regulates the activity of hormone sensitive lipase (HSL) and thereby inhibits lipolysis. This favors lipogenesis and storage of fat. It is known that, in diabetes, insulin's regulation over lipolysis is lost. This results in increased free fatty acids in blood. It is also known that these elevated FFAs impair beta cell function through ceramide production and induce apoptosis. The resulting pancreatic beta cell decompensation worsens the diabetic situation. Interestingly, zinc suppresses phosphorylation of hormone sensitive lipase (HSL), thereby inhibiting free fatty acids release [21]. Zinc deficiency may modify lipid metabolism and membrane integrity and these could impair glucose carrier function [13]. Inhibition of GSK3 by zinc [14] exerts beneficial effects on glycogen metabolism. Also, zinc is reported to inhibit glucagon secretion [22, 23], thus reducing gluconeogenesis and glycogenolysis.

**Figure 2:**
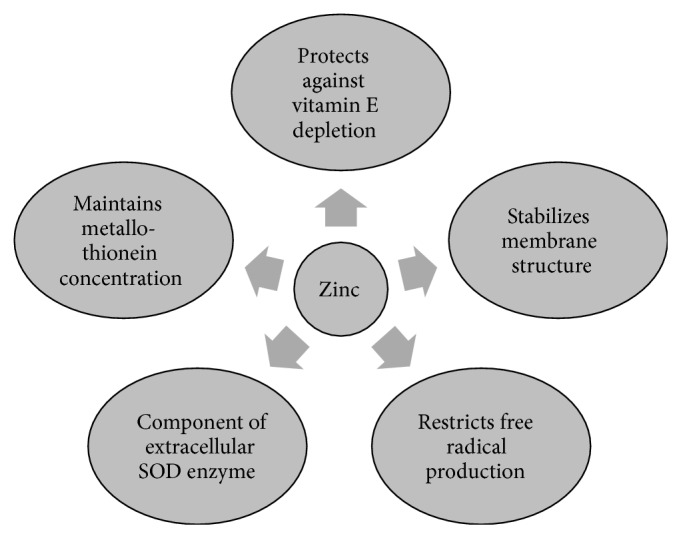
A schematic of the several antioxidant actions of zinc.

Zinc ions also have pronounced effects on redox metabolism, although they are redox inert. Depending on how much zinc is readily available, zinc can either increase the cell's antioxidant capacity or elicit oxidative stress [24]. Zinc contributes to antioxidant defense as a component of Cu-Zn superoxide dismutase and metallothionein. Several mechanisms contribute to the antioxidant effects of zinc [25], summarized in Figure 2.

Zinc also protects beta cells from death, thereby ensuring higher plasma insulin levels. This is achieved by reduced production of interleukins and TNF-*α*, inflammatory mediators of cell death [17]. Ho et al. [26] reported that zinc protected beta cells from oxidative damage and death in streptozotocin and alloxan induced diabetic models.

Thus, zinc plays a pleiotropic role in the maintenance of glucose homeostasis. These metabolic actions of zinc and their mechanism are summarized in Table 2.

Interestingly, it is also known that zinc deficiency coexists with diabetes [12]. Patients with diabetes are more likely to have suboptimal zinc status and a negative correlation has been observed between zinc intake and prevalence of diabetes [27]. Whether zinc deficiency is cause or effect of hyperglycemia is still debatable. Diabetes itself affects zinc homeostasis in many ways resulting in decreases in total body zinc [12]. Zinc deficiency is associated with impairment in glucose tolerance and also an increased sensitivity to diabetogenic agents [26]. Zinc deficiency may also affect the progress of type 2 diabetes. Reduced zinc may also exacerbate the oxidative stress mediated complications of diabetes.

Several preclinical studies have demonstrated the antihyperglycemic effects of zinc supplementation in animal models of type 1 as well as type 2 diabetes [27–29]. Thus, it can be reasoned that a zinc based agent can be used for diabetes therapy. Orally active antidiabetic zinc complexes have also been developed [15, 18, 21, 30, 31]. Despite well proven results in animal models, these zinc complexes are not yet available as antidiabetic therapies in modern medicine.

## 4. Zinc Based Drug in Ayurveda:* Jasada bhasma*

Ayurveda is an ancient Indian system of medicine dating back to 5000 B.C. Ayurveda uses plant-, animal-, mineral-, and metal-based medicines for the treatment of diseases [32–34].* “Rasashastra,*” an integral part of Ayurveda, deals with drugs of mineral origin and details their varieties, characteristics, processing techniques, properties, therapeutic uses, and management of adverse effects in a comprehensive way [35]. It was known in Ayurveda that metals as compared to animal and plant products were not compatible with human body constitution. They could not be consumed in their natural form, hence needed to be processed into fine and soft powder termed “*bhasma*” [36]. The preparation of* bhasmas* includes two main stages:* shodhan* (purification) and* maaran* (incineration).* Shodhan* process involves repeated trituration with herbal extracts, cow urine, milk, ghee, and so forth.* Maaran* process involves repeated cycles of incineration. Thus,* bhasmas* are metals that go through a purification and incineration process that turns them into ash [37, 38].

It was known that incomplete processing would result in metal ion impurities leading to adverse effects and toxicity. Therefore, tests were developed to evaluate the particle size, density, and physical and chemical stability of* bhasmas.* Compliance to these tests indicated complete conversion of metal to oxide form and desired size reduction, thereby implicating safety of the* bhasma* [39]. However, these tests are only qualitative and do not provide information about the chemical composition of* bhasmas* [40]. Pharmaceutical characterization is therefore necessary to identify all the active ingredients in* bhasmas* [41], since most of them are complex herbomineral preparations. Further, standardization of the raw materials, the synthesis procedure, and the finished product is also needed, which ultimately affects the purity, quality, and safety of the* bhasma* [42]. Several modern tools and techniques (namely, electron microscopy, X-ray diffraction, and atomic absorption spectrometry) can be employed to obtain detailed information on the size, structure, and elemental composition of* bhasmas*.

A condition similar to diabetes is recognized in Ayurveda, termed “*Madhumeha*” which means honey-like urine [43, 44]. Ayurvedic treatment of diabetes includes several herbal drugs and also a few mineral preparations including* bhasmas* [45]. In the texts of* Rasashastra* [46],* bhasmas* of* Mandura* (iron),* Vanga* (tin),* Naga* (lead),* Tamra* (copper), and* Jasada* (zinc) have been recommended for the treatment of diabetes. A few scientific reports on the clinical use of* bhasmas* in diabetic patients are available [47, 48].

*Jasada bhasma* (also known as* Yashada bhasma*) is also indicated in various disorders, namely, diabetes, anemia, cough, ulcers, depression, ophthalmic problems, and so forth [49–52]. Standard textbooks on* Rasashastra* recommend* Jasada bhasma* as the treatment of choice for diabetes. However, studies related to pharmacological/clinical investigation of* Jasada bhasma* as antidiabetic agent are few and not comprehensive [53–55]. Clearly, there is a need for undertaking a detailed and systematic study on the proclaimed antidiabetic efficacy of* Jasada bhasma*.

Recent renewed interest in Ayurveda has led to scientific investigations on therapeutic utility of several* bhasmas* [56–62]. Apart from pharmacological validation, pharmaceutical characterization of these* bhasmas* is also necessary. Interestingly, detailed investigation on the composition of* bhasmas* is being carried out by researchers, using modern analytical techniques. Recent reports have suggested the presence of submicronic particles or nanoparticles in* bhasma* preparations. Gold nanoparticles have been detected in* Swarna bhasma* and the formulation is effective antiarthritic agent in rats [63]. Bhowmick et al. [64] reported zinc oxide particles of size ~1 micron in traditionally prepared* Jasada bhasma*. Physicochemical characterization of* Naga* (lead)* bhasma* revealed micron sized particles of lead oxide [65]. Similarly, studies on* Swarna makshika bhasma* revealed that raw* Swarna makshika* is a complex compound that gets converted to simple oxides [66], with micron sized particles [67].

Reports are also available that provide scientific evidence of the incineration cycles during synthesis of* bhasmas*. For example, Wadekar et al. [38] evaluated tin based* bhasma* sample at various stages of calcination and found that the proportion of tin oxide increased with the number of calcination cycles while the particles size stabilized at ~1 micron after the final calcination cycle. Singh and Reddy [68] detected 300 to 500 nm sized particles in* Lauha bhasma*. Further, they observed successive decrease in particle size of* Lauha bhasma* with increase in the number of incineration cycles. In another study using* Lauha bhasma*, it was found that the incineration steps were critical for the formation of nanostructures [69]. Thus, it may be suggested that* bhasmas* contain submicronic particles or nanoparticles of the metal oxide if prepared extremely well.

It is well known that size reduction of particles increases solubility and hence bioavailability. Therefore* bhasmikaran* is expected to reduce the size of metal oxide particles enhancing their bioavailability and bioactivity. Interestingly, in modern science, several researchers have demonstrated enhanced bioavailability of nanoparticles as compared to their bulk form. For example, Ishihara et al. [70] reported higher bioavailability of micronized zinc oxide as compared to standard zinc oxide. In another report, poor water soluble iron compounds when formulated as nanoparticles displayed oral bioavailability similar to soluble salts [71]. Thus, it is safe to assume that* bhasmas* contain nanoparticles that lead to enhanced bioactivity.

## 5. Scientific Studies on* Jasada bhasma *and Its Use in Diabetes Treatment

Over this background of concordance of modern science and Ayurveda, as a case in point, we undertook a systematic study on the physicochemical characterization, antidiabetic efficacy, and safety assessment of* Jasada bhasma* (zinc* bhasma*). In an elaborate study conducted in our laboratory,* Jasada bhasma* was synthesized using traditional method and evaluated for its composition, size, shape, and morphology using several modern physicochemical techniques, namely, high resolution transmission electron microscopy (HRTEM) coupled with selective area electron diffraction (SAED), scanning electron microscopy (SEM) coupled with energy dispersive X-ray spectroscopy (EDS), X-ray diffraction (XRD), and atomic absorption spectrometry (AAS). Efficacy was evaluated using standard pharmacological methods in diabetic rats. Bioavailability and toxicity of* Jasada bhasma* were also assessed in rats.

**Figure 3:**
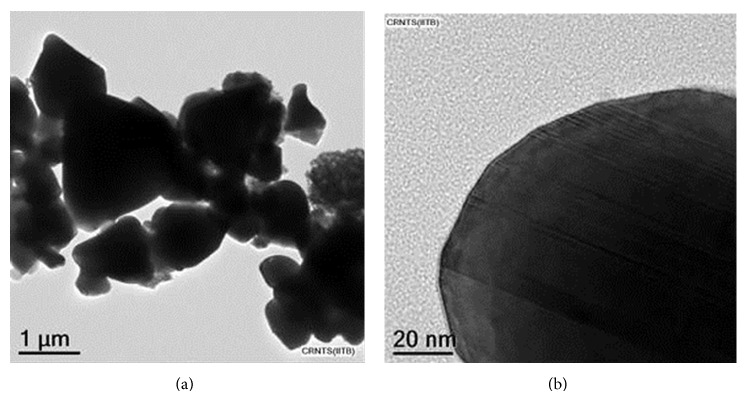
Presence of submicronic particles in* Jasada bhasma*. Images were taken by transmission electron microscopy at low (a) and high (b) magnification.

SEM and HRTEM showed that the traditionally prepared* Jasada bhasma* consisted of 200–500 nm sized particles (Figure 3). EDS, SAED, AAS, and XRD analysis revealed that the preparation consisted predominantly of zinc oxide with hexagonal wurtzite crystal structure (details mentioned in [72]).

Dose range finding studies were carried out in normoglycemic Wistar rats. The dose used in Ayurveda (125 mg, twice a day) led us to simple calculation of 250 mg per day divided by average human body weight of 70 kg, for example, 3.5  mg/kg. To evaluate dose dependent effects, the doses of 3 mg/kg and above were selected. The effective dose range of* Jasada bhasma* in oral glucose tolerance tests was found to be 3–30 mg/kg. For efficacy evaluation,* Jasada bhasma* (1, 3, 10 mg/kg) was administered orally, once daily for 4 weeks, to streptozotocin (STZ) induced type 1 and type 2 diabetic rats. For induction of type 1 diabetes, adult rats were administered 45 mg/kg STZ intravenously whereas, for type 2 diabetes, 5-day-old pups were injected STZ intraperitoneally at the dose of 90 mg/kg.

In case of type 1 diabetic rats,* Jasada bhasma* treatment showed reduction of nonfasted (~20% at 10 mg/kg dose) as well as fasted blood glucose levels (~33% at 10 mg/kg dose). These results were comparable to glibenclamide, used as a positive control. In OGTT,* Jasada bhasma* showed a trend of suppressed glucose levels as well as reduced AUC values (~16%). Treatment also decreased the nonfasted serum insulin levels (~32% at 10 mg/kg dose), suggesting insulin sensitizing effects. In case of type 2 diabetic rats, treatment with* Jasada bhasma* resulted in improved glucose tolerance (~19%), lowered nonfasted (~20%) as well as fasted blood glucose levels (~27%), and reduced serum insulin levels (~27%). These effects were found to be comparable to pioglitazone, a drug widely used clinically.

Next, we evaluated the solubility of* Jasada bhasma* using a simple dialysis experiment. Several researchers have used this technique to estimate nanoparticle dissolution and predict bioavailability. It was found that the dialysability was around 30% in gastric pH, suggesting release of zinc ions. Systemic absorption was assessed by single dose pharmacokinetic study where serum zinc levels were found to be elevated (3.5 folds) after oral administration of* Jasada bhasma* [72].

Since there are lots of concerns related to the toxicity of metallic medicines, we also employed a comprehensive testing strategy for assessment of toxicity profile of* Jasada bhasma*. Cytotoxicity test revealed no loss of cell viability and no effects on cell morphology. Hemolysis was less than 5% (within acceptable limits) after oral administration of* Jasada bhasma* to rats. Acute and subacute toxicity tests demonstrated safety of* Jasada bhasma* up to 300 mg/kg dose in rats [73]. These findings provide concrete scientific evidence that justifies usage of* Jasada bhasma* in diabetes treatment.

## 6. Relevance of* Jasada bhasma* to Zinc Oxide Nanoparticles Based Drug

Encouraged by the results obtained in case of* Jasada bhasma*, we extended our work towards the development of a zinc based antidiabetic agent for modern medicine. Since our studies clearly demonstrated the presence of submicronic zinc oxide particles in* Jasada bhasma*, we hypothesized that nanoparticles of zinc oxide (≤10 nm size) should also be able to exert antidiabetic effects. An elaborate study was undertaken to investigate this possibility [74, 75].

In proof of concept studies, single administration of zinc oxide nanoparticles resulted in significant suppression of glucose levels in OGTT carried out in both type 1 and type 2 diabetic rats (~22% and ~30%, resp.). These effects appeared to be more prominent than those obtained with similar doses of* Jasada bhasma*. After 4 weeks of treatment (1, 3, and 10 mg/kg doses) to diabetic rats, significant reduction in glucose levels was seen in both nonfasted (~19% and ~29% in type 1 and type 2 diabetic rats, resp.) and fasted state (~26% and ~21% in type 1 and type 2 diabetic rats, resp.), suggesting multiple mechanisms. Reduction of nonfasted glucose levels can be attributed to insulin secretagogue effects. Reduction of fasted glucose levels may be due to glucagon inhibition, as is reported with zinc [22, 23]. Increased serum insulin levels (~35% and ~70% in type 1 and type 2 diabetic rats, resp.) suggested insulin secretagogue effects. Reduction in serum TG (~48%) and FFA (~41%) levels was also observed after treatment indicating beneficial effects on lipid metabolism. Overall results suggested that zinc oxide nanoparticles were more potent and efficacious than* Jasada bhasma* [74].

Differences in efficacy of* Jasada bhasma* (200–500 nm particles) and zinc oxide nanoparticles (≤10 nm) suggested possible size dependent differences in bioavailability. Dialysability experiments revealed better dissolution and release of zinc ions from zinc oxide nanoparticles (~40%) as compared to* Jasada bhasma* (~30%). Further, it was found that >50% of the total zinc was left undialyzed. These results suggested that under* in vivo* conditions,* Jasada bhasma,* and zinc oxide nanoparticles are encountered as zinc ions as well as particulates, after oral administration [74].

The soluble fraction of* Jasada bhasma* and zinc oxide nanoparticles could get immediately absorbed from the intestine and result in initial spurt of zinc in blood. The particulates could be taken up by enterocytes through size limited endocytosis. It could be expected that the uptake of particulate fraction of zinc oxide nanoparticles from the intestine would be higher than that of* Jasada bhasma*. In circulation, very small particles (1–20 nm) can slowly extravasate into the interstitial spaces and then can be taken up by tissue cells [76]. Once in cells, particles encounter increasing acidic environment as they move from early to late endosomes and finally to lysosomes, resulting in dissolution of particulates [77]. Particulate component would thus result in slow and continuous release of zinc ions, acting as a depot.

As predicted, pharmacokinetic evaluation showed that serum and tissue zinc levels in zinc oxide nanoparticles treated rats were higher than* Jasada bhasma* treated rats. Further, serum zinc levels were maintained for 24 h in zinc oxide nanoparticles treated rats, whereas they declined within 4 h in* Jasada bhasma* treated rats. Long circulation of zinc oxide nanoparticles as compared to* Jasada bhasma* could possibly increase chances of their passage to tissues and hence higher cellular uptake, corresponding to the observed higher tissue zinc levels. These results correlated with* in vivo* efficacy studies where zinc oxide nanoparticles displayed a more potent and efficacious antidiabetic profile than* Jasada bhasma* [74].

Next,* Jasada bhasma* and zinc oxide nanoparticles were investigated in rat insulinoma (RIN5f) cell line to elucidate the possible mechanism of antidiabetic activity.* Jasada bhasma* did not enhance insulin secretion, whereas zinc oxide nanoparticles resulted in dose- and glucose- dependent insulin secretagogue effects. These results suggested that* Jasada bhasma* had poor cell permeability. Further, zinc oxide nanoparticles treatment* per se* enhanced SOD activity of RIN5f cells and also protected cells against H_2_O_2_ induced oxidative stress, suggesting antioxidant effects. Since uncontrolled hyperglycemia and oxidative stress contribute to development of diabetic complications, zinc oxide nanoparticles by virtue of antihyperglycemic and antioxidant effects may be expected to delay the progression of disease and development of associated complications [74].

Several toxicity tests were then performed to evaluate the safety of zinc oxide nanoparticles. Cytotoxicity was not seen up to 10 *µ*g/mL concentrations of zinc oxide nanoparticles (concentrations resulting in insulin secretion) in RIN5f cells. Further to evaluate genotoxic effects of zinc oxide nanoparticles,* in vivo* micronucleus test was performed using Wistar rats. Micronuclei formation was not increased after zinc oxide nanoparticles treatment, indicating no risk of genotoxicity [75].

Since significant antihyperglycemic activity was seen at 3 mg/kg dose, toxicity studies were performed at 30 and 300 mg/kg, for example, 10 and 100 times the effective dose. In acute toxicity study, no behavioral abnormality or clinical signs or mortality was recorded after zinc oxide nanoparticles treatment at 300 mg/kg dose. Further, no effect was seen on body weight and major organ weights and tissue histology. In subacute toxicity test, 28 days of treatment with zinc oxide nanoparticles at two doses, 30 and 300 mg/kg, did not result in any significant effects on body weight gain or organ weight to body weight ratios. SGOT and SGPT activities and creatinine and urea levels were not altered in treatment groups as compared to control group, indicating no major organ damage. Histological examination of tissue sections did not reveal any necrotic damage. Taken together, toxicity studies revealed the safety of zinc oxide nanoparticles up to 100 times the effective dose [75].

Overall, it was clearly evident from our studies that zinc oxide nanoparticles can elicit potent antidiabetic activity in type 1 and type 2 diabetic rats [75]. Thus, taking inspiration from the usage of* Jasada bhasma* in diabetes, a new chemical entity (zinc oxide nanoparticles) is proposed in modern medicine warranting further investigation.

## 7. Conclusions and Future Prospects

The roles of metals in human physiology are well established. It is also known that many metals are required in trace amounts for normal metabolism and their deficiency leads to diseases. In Ayurveda, metal-based preparations, for example,* bhasmas*, are indicated for the treatment of several diseases. However, in present day Ayurvedic practice, the use of* bhasmas* is limited. This could be because the synthesis procedures of* bhasmas* are laborious, time consuming, and often difficult to interpret from ancient texts. Different protocols exist to get several types of* bhasma* of the same metal. Hence, selection of the synthesis protocol requires sound knowledge of the Ayurveda system. To address this issue, standardization of the synthesis procedure and its detailed documentation would be helpful.

Standards for manufacture and quality control are not yet properly defined and enforced. Development of detailed testing strategy using modern analytical tools would be useful for ensuring quality of* bhasmas*, especially absence of heavy metal impurities. FDA regulations for sale of* bhasmas* need to be clearly defined and executed to avoid the use of substandard drugs.

Parallely, scientific studies on all the* bhasmas* described in Ayurvedic texts are needed. Pharmacological validation studies can be undertaken to generate evidence of the efficacy of* bhasmas*. Further, detailed investigation of the mechanism of action using modern research tools (namely, proteomics and genomics) will help solve the mystery of the observed effects of these Ayurvedic medicines. For example, a report on* Rasa-Sindoor* (mercury and sulfur) has detailed the various mechanisms by which it exerts a holistic effect in neurodegenerative disorders such as Huntington's and Alzheimer's disease [78]. Moreover, Ayurvedic practitioners should be encouraged to publish reports of patient outcomes. Such reports will add the much needed clinical evidence of the utility of* bhasmas*.

Most importantly, the knowledge gained out of Ayurvedic texts and evidence based studies should be extended to modern medicine. Although there are several drugs available for diabetes in the market, none of them is free from adverse effects. On the other hand, Ayurvedic medicines, namely,* bhasmas,* are known to be effective at very low doses and devoid of toxic effects. Once the active ingredients of* bhasmas* are identified, these metal oxides can be synthesized and evaluated as a new chemical entity in modern drug discovery. Taking inspiration from the fact that* bhasmas* contain submicronic or nanoparticles that enhance bioavailability, metal based nanomedicines can be developed for diabetes. This would help in utilizing the age old wisdom of Ayurveda for the development of newer drugs in modern medicine.

## Figures and Tables

**Table 1:** Summary of the current therapeutic agents for type 2 diabetes and associated side effects.

Drug class/agent	Drug effect/action	Side effects/demerits
Metformin	Suppresses hepatic glucose output	Lactic acidosis and GI problems
Insulin secretagogues (sulphonylureas)	Increase insulin secretion	Hypoglycemia and weight gain
PPAR*γ* agonists (thiazolidinediones)	Increase insulin sensitivity	Peripheral edema, weight gain, and anemia
Alpha-glucosidase inhibitors (acarbose)	Inhibit glucose absorption	Loose stools and flatulence
GLP-1 analogues (liraglutide)	Increase glucose stimulated insulin secretion	Patient compliance in case of peptide analogues (injection)
DPP-IV inhibitors (sitagliptin)	Enhance endogenous GLP-1 action	Specificity issues
SGLT2 inhibitors (canagliflozin)	Inhibition of glucose reabsorption in kidneys	Urinary tract infections

**Table 2:** A summary of metabolic actions of zinc and their mechanism.

Metabolic action	Mechanism of action	References
Increases insulin action	Increases stability and receptor binding	[11, 12]
Improves insulin signaling	PTP1B inhibition	[16]
Enhances insulin signaling	Increases receptor phosphorylation and PI3K activity	[17]
Beneficial effect on glycogen metabolism	GSK3 inhibition	[14]
Increases glucose uptake	GLUT4 translocation in adipocytes	[19, 20]
Decreases lipolysis	Inhibition of HSL and FFA release	[21]
Inhibits glucagon secretion	Opening of K_ATP_ channels in pancreatic alpha cells	[22, 23]
Inhibits intestinal glucose absorption	Inhibits alpha-glucosidase enzyme	[18]
Reduces oxidative stress	Enhances SOD activity	[25]
Protects beta cells	Modulates NF*κ*B activation	[26]
